# Low-Loss and Stable Light Transmission in Nano-Core Plus Node-Free Anti-Resonant Hollow-Core Fiber

**DOI:** 10.3390/nano15181458

**Published:** 2025-09-22

**Authors:** Yuyi Yin, Tingwu Ge, Tong Zhang, Zhiyong Wang

**Affiliations:** School of Physics and Optoelectronic Engineering, Beijing University of Technology, Beijing 100124, China; yinyuyi@emails.bjut.edu.cn (Y.Y.);

**Keywords:** NPNANF, transmission loss, optical confinement, high-power communication, anti-resonant hollow-core fiber

## Abstract

Anti-resonant hollow-core fibers (AR-HCFs) are emerging as highly promising candidates for high-power laser transmission and low-loss optical communication. Despite their advantages, issues such as scattering loss and core-mode instability remain significant obstacles for their practical implementation. In this study, we propose a novel hybrid fiber structure, the nano-core plus node-free anti-resonant hollow-core fiber (NPNANF), which integrates a solid, high-index nano-core within a six-tube node-free anti-resonant cladding. This hybrid design effectively enhances optical confinement while minimizing scattering losses, without relying solely on anti-resonant guidance. Numerical simulations employing the beam propagation method (BPM) and finite element analysis (FEA) demonstrate that an optimal nano-core diameter of 600 nm leads to a remarkable reduction in transmission loss to 0.025 dB/km at 1550 nm, representing a 99.8% decrease compared to conventional NANF designs. A comprehensive loss model is developed, incorporating contributions from confinement, scattering, and absorption losses in both the hollow cladding and the solid core. Parametric studies further illustrate the tunability of the fiber’s design for various high-performance applications. The proposed NPNANF achieves an ultra-low transmission loss of 0.025 dB/km, representing a >99.8% reduction compared to conventional NANF, while confining more than 92% of optical power within the nano-core. Its resistance to bending loss, strong modal stability, and balance between hollow-core and solid-core guidance highlight the advantages of NPNANF for long-haul optical communication and high-power photonics.

## 1. Introduction

Hollow-core fibers (HCFs) have attracted significant attention due to their numerous advantages, including low optical nonlinearity, ultra-low Rayleigh scattering, minimal chromatic dispersion, reduced latency, and low optical attenuation. These features make them ideal for a wide range of applications, such as high-power laser delivery, low-latency optical communication, and gas sensing [1,2,3,4]. Current research on HCFs primarily focuses on minimizing transmission loss, particularly through structural optimization in two major types of fibers: hollow-core photonic bandgap fibers (HC-PBGFs) and hollow-core anti-resonant fibers (HC-ARFs). The development of HC-PBGFs dates back to 1995, with early designs based on periodic hollow cores [5,6]. By the early 2000s, the transmission loss of HC-PBGFs had been reduced to 1.7 dB/km in the telecommunication band [7]. However, due to surface scattering limitations (SSL), further loss reduction stagnated at around 1.2 dB/km for decades [7].

Beyond current optical communication networks, hollow-core fibers (HCFs) offer advantages such as ultralow Rayleigh scattering, low latency, and negligible nonlinearities, making them strong candidates for future 6G optical backhaul links, ultra-high-capacity data center interconnects, and integration with reconfigurable intelligent surface (RIS)-assisted wireless systems [8]. These features demonstrate the potential of HCF technology as a scalable fiber-based backhaul solution, complementing free-space and wireless links, and serving as a promising candidate for 6G networks and data center interconnections. [8].

In parallel, alternative designs were explored, such as Kagome fibers [9,10], which utilized a different guiding mechanism and led to the development of HC-ARFs based on anti-resonant theory. Despite simpler geometries like single-ring tubular or ice-cream-shaped structures [11], these fibers still exhibited high losses (≥50 dB/km). To address this, innovations such as node-free designs of HC-ARFs achieved transmission losses as low as 7.7 dB/km at 1550 nm [12]. However, these fibers still fall short of the ultra-low-loss requirements for modern optical communication systems.

To overcome the limitations of existing designs, the nested anti-resonant node-free fiber (NANF) has emerged as a highly promising solution, reporting losses between 0.22 and 1.3 dB/km [13,14]. By embedding smaller capillaries inside the primary cladding tubes, NANFs enhance anti-resonance and suppress scattering loss. However, these designs still face challenges such as higher-order mode loss and structural instability, particularly with five-ring geometries that induce stress concentration [15,16]. In contrast, six-ring geometries provide better mechanical stability and are more suited for high-power applications [17].

In this work, we propose a nano-core plus nested anti-resonant node-free fiber (NPNANF) structure to improve both transmission efficiency and output stability. The proposed design builds upon the classic six-ring NANF geometry, with additional dimensional optimization and a central nano-core to enhance field confinement and reduce scattering. The nano-core supports evanescent field concentration, allowing for the light leakage to be effectively recycled into the intermediate air region via the anti-resonant cladding. Finite element analysis (FEA) is employed to study the influence of nano-core diameter on transmission loss at 1550 nm. Our simulation results reveal that a 600 nm nano-core diameter yields the best performance, achieving a transmission loss of 5 dB/km, which represents a 75% reduction compared to a conventional hollow-core design with the same geometry.

Additionally, the NPNANF demonstrates low bending loss and stable light propagation, making it a strong candidate for long-haul transmission systems, high-power laser delivery, and precision gas-sensing applications.

## 2. Performance Analysis of NPNANF

### 2.1. Structure Design of NPNANF

**Figure 1:**
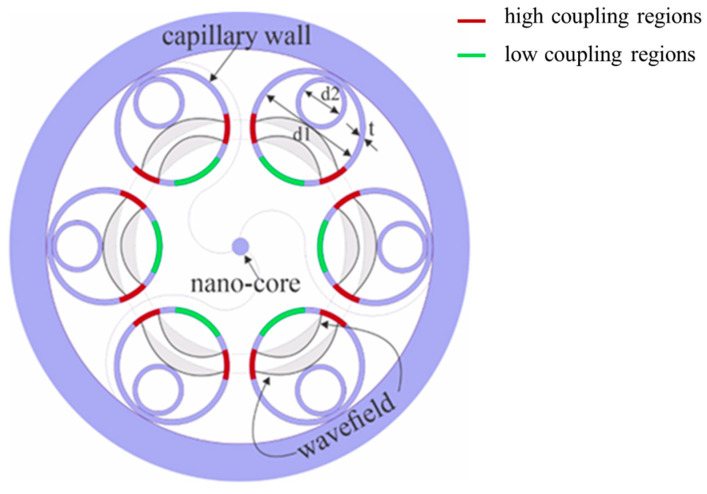
Illustration of structural design of proposed NPNANF.

The structural design of the proposed NPNANF is illustrated in Figure 1. This fiber consists of a six-ring cladding structure, where each ring is composed of nested hollow silica tubes that act as anti-resonant barriers to confine light. At the fiber center, a solid nano-core of diameter 600 nm is introduced to enhance light confinement, significantly improving transmission efficiency. The nano-core is supported by two semicircular silica sheets that provide mechanical stability while maintaining high transparency.

Surrounding the core is an intermediate air layer and a set of densely packed air holes arranged in a hexagonal lattice, occupying approximately 20% of the overall fiber cross-section. These features form an anti-resonant guiding layer, which minimizes the overlap between the optical field and the solid material, thereby reducing scattering and confinement losses [17]. Furthermore, the intermediate air layer acts as an optical barrier, reflecting stray light and reinforcing confinement within the core.

The wall thickness of each nested tube (denoted as t) is set to 1 μm, ensuring efficient anti-resonant reflection. The key geometrical parameters of the proposed NPNANF are summarized in Table 1.

The design enables strong light confinement in the nano-core while maintaining low-loss and stable single-mode propagation. Additionally, the use of multiple nested air-hole layers serves to suppress higher-order mode (HOM) transmission and enable low-loss multimode guiding through mode isolation [18]. This structural configuration contributes to exceptional optical properties, including low dispersion, low nonlinearity, and low latency across multiple transmission bands [19].

In summary, the integration of a nano-core with a six-ring nested anti-resonant cladding provides a compact yet effective approach to achieving stable and efficient light guidance, making the NPNANF a promising candidate for next-generation high-performance optical fiber systems.

### 2.2. Working Principle of the NPNANF

The guiding mechanism of NPNANF is hybrid in nature: light couples into the nano-core and partially leaks into the surrounding air gap, but the anti-resonant cladding tubes reflect this leakage back toward the core. This recycling process strengthens confinement, reduces scattering loss, and ensures low-loss transmission with stable modal behavior.

**Figure 2:**
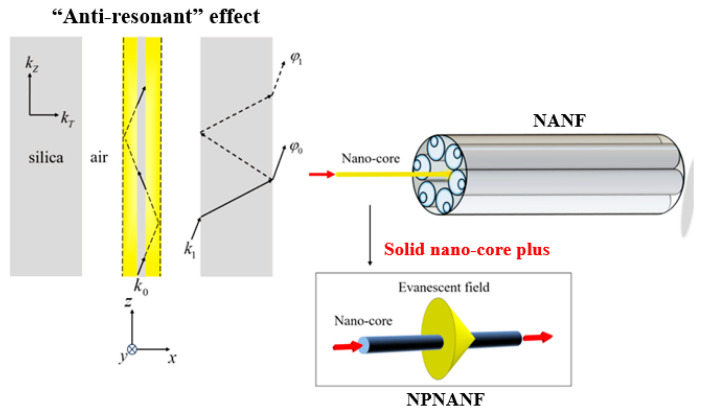
Schematic of light propagation mechanism in NANF and NPNANF.

The operating principle of NPNANF is based on the anti-resonant guiding mechanism, wherein light propagates within the nano-core and partially leaks into the surrounding region, but is effectively reflected and confined by the anti-resonant cladding tubes. These tubes function as Fabry–Pérot cavities, reflecting light back into the core and suppressing optical leakage. Unlike conventional NANF, the introduction of a nano-core allows for evanescent field coupling into the intermediate air region, which is then reflected back into the core by the anti-resonant cladding. This unique feature helps reduce scattering losses and enhances optical confinement, improving overall transmission efficiency. A schematic diagram illustrating the light propagation mechanism in both conventional NANF and the proposed NPNANF is shown in Figure 2.

When the light beam is incident on the core-cladding interface with a glancing angle, the longitudinal propagation constant $k_{Z}$ can be approximated by $n_{0}k_{0}$. Thus, the angle of incidence is approximately equal to 90°. By using Snell’s law, the transverse propagation constant in the silica layer can be expressed as $k_{T}=k_{0}\sqrt{n^{2}-n_{0}^{2}}$. The thin wall of the silica tube can be considered as a cavity in this case. When the light beam travels through the silica tube with refraction and reflection, a phase difference $\varphi_{1}-\varphi_{0}\approx 2tk_{T}$ will be generated. Under the condition, where $\varphi_{1}-\varphi_{0}=\left(2m-1\right)\pi$, the two light beams will interfere with deconstructivity. Hence, the relationship between light wavelength and wall thickness of silica tube can be rewritten as $\lambda_{m}=\frac{2t}{m}\sqrt{n^{2}-1}$, where m is the resonance order (non-zero integer); $\lambda_{m}$ indicates the incident wavelength, and n is the refractive index of the cladding glass.

In contrast to conventional hollow-core anti-resonant fibers, the NPNANF introduces a central nano-core, which allows part of the light field to couple into the intermediate air region in the form of evanescent waves. These waves are then reflected back by the anti-resonant cladding, enabling quasi-bound oscillation between the core and cladding regions. This significantly reduces scattering loss and enhances confinement.

To further understand the field distribution, the planar waveguide model (shown in Figure 2) is used, particularly when the wavelength λ is smaller than the nano-core diameter *d*. For incident angles greater than the critical angle, total internal reflection occurs. However, due to refractive index discontinuity, the reflected beam experiences Goos–Hänchen displacement, effectively increasing the optical path. This leads to the mode field being distributed not only in the core, but also in the cladding as evanescent waves, particularly pronounced when *d* approaches the sub-micron scale.

The mode field distribution $\psi_{0}\left(d,\theta_{0}\right)$ in such waveguides can be expressed as

(1) $$\psi_{0}\left(d,\theta_{0}\right)=AJ_{v}\left(U\right)\cos v\;\theta_{0}$$

(2) $$\psi_{0}\left(d,\theta_{0}\right)=A\frac{J_{v}\left(U\right)}{K_{v}\left(W\right)}K_{v}\left(iW\right)\cos v\;\theta_{0}$$

where *A* is the coefficient, and *θ*_0_ is the azimuth angle. Equations (1) and (2) describe the case when *d* ≤ *λ* and *d* > *λ* (incident wavelength), respectively. More specifically, the light transmission characteristics of NPNANF are greatly influenced by the diameter of the nano-core. A larger core diameter results in the restriction of the light field within the core, propagating along the solid nano-core with minimal field outside the core, thereby diminishing the effectiveness of the anti-resonance layer. In contrast, a reduction in core diameter leads to the leakage of the light field into the surrounding anti-resonance layer. The unique structure of the anti-resonance layer leads to partial light reflection and confinement back within the nano-core, enabling lightwaves to oscillate between the hollow cores and the nano-core. These anti-resonance characteristics effectively reduce scattering losses, facilitating low-loss transmission.

Furthermore, the intermediate air layers between the nano-core and cladding function as optical barriers or “traps,” reflecting stray light back toward the nano-core. This mechanism strengthens light confinement and improves transmission efficiency, making the NPNANF ideal for low-loss, long-haul communication and high-power laser delivery applications.

The anti-resonant transmission condition is determined by the phase relationship of light traversing the thin silica wall of thickness, where each tube functions as a Fabry–Pérot cavity. The phase difference between forward and backward waves is

(3) $$\phi_{1}-\phi_{0}\approx 2t\sqrt{k_{0}^{2}n^{2}-k_{0}^{2}}=2tk_{T}$$

Anti-resonance occurs when $\phi_{1}-\phi_{0}=\left(2m-1\right)$π, yielding

(4) $$\lambda_{m}=\frac{2t}{m\sqrt{n^{2}-1}}$$

where *t* is the silica wall thickness; *m* is the resonance order, and *n* is the refractive index of silica. By selecting *t* ≈ 1 μm, we ensure efficient guidance with low confinement loss in the C- and L-bands (1530–1625 nm).

**Figure 3:**
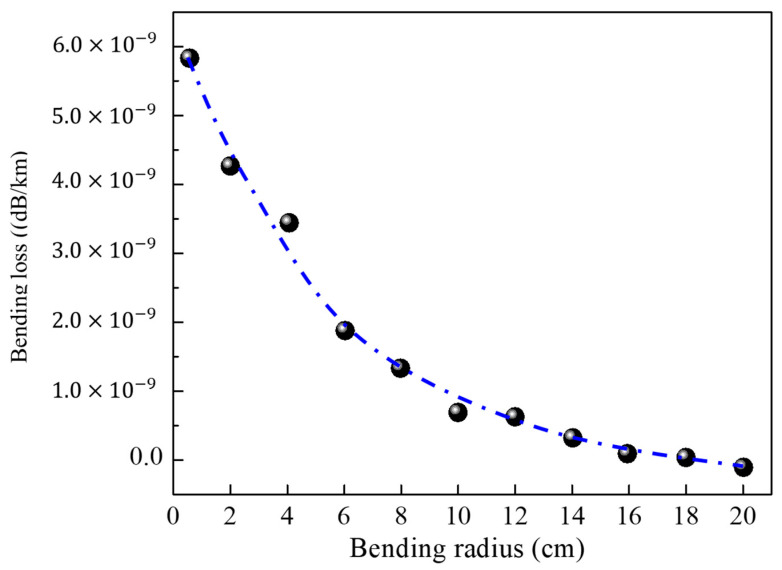
Bending loss of NPNANF as a function of bending diameter.

In the first stage, the bending loss performance concerning the NPNANF and NANF with the same dimensions has been studied, as shown in Figure 3. It is seen that NPNANF is quite resistant to bending with acceptable bending loss, with a moderate increase in bending loss toward a smaller bending radius (in the order of 10^−9^). It is apparent that the light field transmission of NPNANF is relatively stable compared to other types of hollow-core fibers. With the further stabilization of anti-resonance and optical “trap” effect, high transmission efficiency and low transmission loss are obtained by NPNANF, which is particularly suitable for applications such as high-power laser transmission and long-distance optical communication systems, where transmission efficiency and signal integrity are of great importance.

Table 2 summarizes the bending loss performance of various hollow-core fiber designs in recent years. While the proposed NPNANF still exhibits relatively high bending loss in tight-radius conditions, its overall transmission efficiency and mode stability are superior and can be further improved through optimization.

This hybrid guiding mechanism allows NPNANF to transmit light efficiently by combining strong nano-core confinement with anti-resonant cladding reflections that recycle evanescent leakage. The six-ring cladding ensures mechanical robustness, while the nano-core provides stable mode confinement, making the system suitable for long-term stable optical devices such as high-power laser delivery fibers and backhaul optical links.

### 2.3. Simulation of Transmission Properties of NPNANF

Numerical simulations employed the Beam Propagation Method (BPM) in RSoft and Finite Element Analysis (FEA) in COMSOL version 6.3. For BPM, a computational domain of 60 × 60 μm^2^ with perfectly matched layers (PML) at all boundaries was used; mesh step size was refined to 0.05 μm near the nano-core. For FEA, the domain was enlarged to 80 × 80 μm^2^, with second-order absorbing boundary conditions to minimize reflections and ensure convergence.

**Figure 4:**
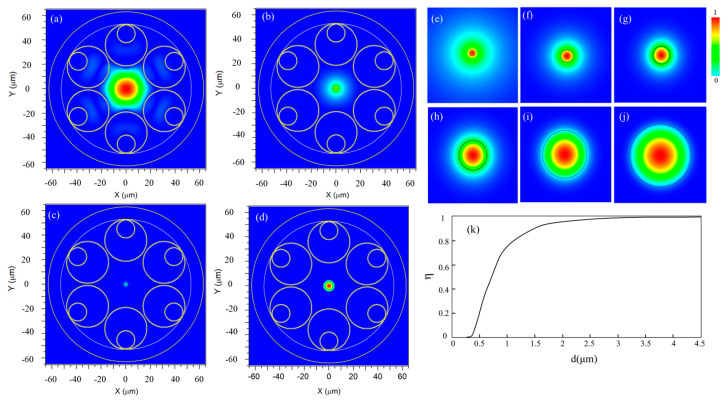
Energy field diagrams of different core diameters: (**a**) hollow-core, namely NANF; (**b**) 0.2 μm; (**c**) 0.6 μm; and (**d**) 10 μm. (**e**–**j**) Energy field distribution at 1550 nm for different solid nano-core sizes at 0.4, 0.6, 0.8, 1, 2, and 4 μm. (**k**) Variation curve of energy percentage η as a function of nano-core diameter d of NPNANF.

To evaluate the transmission performance of the proposed NPNANF structure, numerical simulations were carried out using the Beam Propagation Method (BPM) implemented in RSoft software version 2020. A key factor influencing the transmission behavior is the energy distribution of the incident light within the nano-core, which is highly dependent on the core diameter. Figure 4 shows the two-dimensional electric field distributions under varying nano-core diameters. As illustrated in Figure 4a–d, the extent of evanescent field penetration and the corresponding confinement within the core are significantly influenced by the core size.

BPM simulations used a 60 × 60 μm^2^ domain with PML boundaries, mesh step 0.05 μm near the nano-core. FEA simulations extended to 80 × 80 μm^2^ with second-order absorbing boundaries to ensure convergence.

As the nano-core diameter increases, a greater portion of the optical field becomes confined within the core region. For example, at 0.6 μm, the optical field exhibits strong confinement while maintaining a balance that still leverages the anti-resonance effect from surrounding cladding tubes. In contrast, smaller diameters (e.g., 0.2–0.4 μm) lead to broader evanescent fields that extend into the surrounding anti-resonant air layers, reducing mode confinement. However, the anti-resonant structure partially reflects and recaptures these evanescent components, thereby maintaining effective transmission with reduced scattering loss. The field distributions presented in Figure 4e–j demonstrate this phenomenon more clearly, showing how the mode profile evolves with different core sizes under a fixed wavelength of 1550 nm. A black contour is used to indicate the nano-core boundary. As the core diameter increases, the confinement becomes stronger, minimizing energy leakage into the cladding.

To quantify the impact of core size on transmission confinement, the energy confinement ratio *η*, defined as the ratio of optical energy within the nano-core to the total guided energy, is plotted in Figure 4k as a function of the nano-core diameter *d*. When *d* exceeds 3 μm, nearly all optical energy is confined within the nano-core, indicating a transition from anti-resonant waveguiding to a solid-core dominated guidance regime.

(5) $$\eta =\frac{\int_{S_{\mathrm{core}}}|E{|}^{2}dS}{\int_{S_{\mathrm{total}\;}}|E{|}^{2}dS}$$

where $S_{\mathrm{core}\;}$ is the nano-core cross-section, and $S_{\mathrm{total}\;}$ is the entire fiber cross-section. Simulations assume continuous-wave excitation at 1550 nm and single-mode operation.

These simulation results confirm the theoretical predictions outlined in Section 2.2 and validate that the optimal nano-core diameter balances both evanescent wave interaction and energy confinement. Particularly, a core size of 0.6 μm was found to deliver the best trade-off between low-loss transmission and strong optical confinement, offering a practical route for high-performance applications.

All simulations launched the ${HE}_{11}$ fundamental mode with a Gaussian-like intensity profile, normalized input power of 1 W, and linear x-polarization. These excitation conditions were kept identical across all geometric parameter sweeps to ensure consistent comparison.

## 3. Loss Analysis of NPNANF

### 3.1. Loss Mechanism

In order to evaluate the practical viability of the proposed Nano-Core Plus Node-Free Anti-Resonant Hollow-Core Fiber (NPNANF) for high-power laser delivery and long-haul optical communication, a detailed analysis of its optical loss mechanisms is essential. For conventional node-free anti-resonant fibers (NANFs), the total transmission loss consists of three primary components: confinement loss (*CL*), scattering loss (*SL*), and absorption loss (*AL*). The total loss *f*(*r*) of a NANF fiber can be expressed as

(6) $$f\left(r\right)=CL_{H}+SL_{H}+AL_{H}$$

where $CL_{H}$, $SL_{H}$, and $AL_{H}$ represent the confinement, scattering, and absorption losses in the hollow core, respectively.

In contrast, the introduction of a solid nano-core in the NPNANF fiber alters these loss mechanisms significantly. As shown in previous results (see Figure 4), the light propagation within NPNANF is predominantly confined to the solid nano-core, rather than relying entirely on the anti-resonant cladding for light confinement. This modification fundamentally changes the optical loss profile, such that the primary contributors to the total loss are scattering and absorption within the nano-core. Therefore, the total loss $g\left(r\right)$ in NPNANF can be described as

(7) $$g\left(r\right)=SL_{S}+AL_{S}$$

where $SL_{S}$ and $AL_{S}$ represent the scattering and absorption losses within the solid nano-core, respectively. Rayleigh scattering in the nano-core is particularly prominent due to its high surface-area-to-volume ratio.

The structural configuration of NPNANF combines both hollow-core (anti-resonant) and solid-core (nanowire) elements, with light waves interacting with both components during transmission. While the anti-resonant structure of the hollow-core fiber serves to minimize scattering and absorption in the cladding, some amount of light inevitably leaks into the cladding region. This leakage results in confinement loss, scattering loss, and absorption loss in the hollow cladding, while the solid nano-core primarily contributes scattering loss (due to Rayleigh scattering) and absorption loss due to material properties.

Thus, the total loss of the NPNANF fiber, combining both hollow-core and nano-core components, can be expressed as the sum of the two contributions:

(8) $$\begin{array}{c} \alpha \left(\lambda \right)=f\left(r\right)+g\left(r\right)\\ =\left(\frac{40\pi \cdot \mathrm{lm}\left(n_{\mathrm{eff},\;H}\right)}{\lambda \cdot \ln(10)}+\frac{A_{R,H}}{\lambda^{4}}+\alpha_{d,H}\cdot \frac{\int_{S_{d,H}}p_{z}dS}{\int_{S_{\infty ,H}}p_{z}dS}\right)+\left(\frac{A_{R,S}}{\lambda^{4}}+\alpha_{d,S}\cdot \frac{\int_{S_{d,S}}p_{z}dS}{\int_{S_{\infty ,S}}p_{z}dS}\right) \end{array}$$

where $\alpha \left(\lambda \right)$ is the total loss; *f*(*r*) is the hollow-core loss; *g*(*r*) is the solid-core nanowire loss; $A_{R,S}$ is the Rayleigh scattering coefficient of the nanowire solid core; *λ* represents the scattering properties of the material; $\alpha_{d,H}$ is the impurity absorption coefficient of the hollow-core fiber cladding per unit length; $\int_{S_{d,H}}p_{z}dS$ is the product of the power flux density along the *z*-axial direction within the impurity region $S_{d,H}$, and $\int_{S_{\infty ,H}}p_{z}dS$ is the integral of the power flux density along the axial *z*-direction over the entire fiber cross-section $S_{\infty ,H}$. To achieve total loss minimization, a simple and direct method is to modify the dimensions of NPNANF, especially tuning the section ratio between the hollow core and nano-core to reach the loss balance. Following the rule, the optimal geometric parameters of NPNANF could be realized with the balance of the diameter of the solid nano-core and hollow core. To minimize the total loss *α*(*λ*), both the hollow-core loss *f*(*r*) and the nano-core loss *g*(*r*) should be optimized simultaneously. For the loss accumulated in hollow core *f*(*r*), it can be effectively reduced via increasing the air hole size, improving the material purity in the cladding part, and optimizing the anti-resonance structure. Consequently, the minimum transmission loss for the hollow core part could be written as

(9) $$\min f\left(r\right)=\min\left(CL_{H}+SL_{H}+AL_{H}\right)$$

More specifically, increasing the air hole size helps in reducing confinement loss, while material optimization helps decrease scattering and absorption losses. In terms of the solid nano-core loss $g\left(r\right)$, it mainly consists of scattering loss ($SL_{S}$) and absorption loss ($AL_{S}$). As explained, Rayleigh scattering ($SL_{S}$) is more prominent due to the large surface area. This could be reduced by improving the surface quality (e.g., smoothness) and reducing dimensional fluctuations. On the other hand, absorption loss $AL_{S}$ could be effectively controlled by improving the material purity. Therefore, with all efforts applied to the nano-core design, the nano-core loss $g\left(r\right)$ could reach its minimum, as expressed by

(10) $$\min g\left(r\right)=\min\left(SL_{S}+AL_{S}\right)$$

By adjusting the fiber’s geometric parameters and material properties, the optimal total loss of NPNANF could be achieved with the best performance of hollow core and solid nano-core:

(11) $$\begin{array}{c} {\alpha \left(\lambda \right)=\min\left(f\left(r\right)+g\left(r\right)\right)}_{min}\\ =\min\left(\frac{40\pi \cdot \mathrm{lm}\left(n_{\mathrm{eff},\;H}\right)}{\lambda \cdot \ln(10)}+\frac{A_{R,H}}{\lambda^{4}}+\alpha_{d,H}\cdot \frac{\int_{S_{d,H}}p_{z}dS}{\int_{S_{\infty ,H}}p_{z}dS}+\frac{A_{R,S}}{\lambda^{4}}+\alpha_{d,S}\cdot \frac{\int_{S_{d,S}}p_{z}dS}{\int_{S_{\infty ,S}}p_{z}dS}\right) \end{array}$$

From Equation (8), it is obvious that the total loss arises from the mutual effects of the solid nano-core and hollow core sessions. When a light beam is incident on NPNANF, different types of loss mechanisms arise. Therefore, the optimization process includes the structural design and material selection for both NANF and nano-core parts. In the following section, the detailed theoretical calculation on reducing the total transmission loss will be presented, which is proven as an effective strategy for fiber loss reduction and hollow core-based structural optimization.

### 3.2. Optimization of Losses in Hollow-Core and Nano-Core Components

The diameter of the nano-core plays a critical role in determining the optical performance of the proposed NPNANF. It not only influences the confinement loss but also governs the overall transmission efficiency and modal behavior of the fiber. Particularly, variations in the core diameter affect the coupling strength between anti-resonant tubes and may even trigger Fano-like interference effects.

The hollow-core component loss $f\left(r\right)$ is predominantly influenced by the confinement, scattering, and absorption losses in the cladding. The following strategies are employed to minimize these losses:

(1) Increasing Air Hole Size: Enlarging the air holes in the hollow-core fiber can help reduce confinement loss by increasing the effective refractive index contrast between the core and cladding, thereby improving the anti-resonance effect;
(2) Improving Material Purity: Reducing impurities in the cladding material helps minimize scattering and absorption losses, contributing to the overall reduction in total transmission loss;
(3) Optimizing Anti-Resonance Structure: Refining the anti-resonant cladding design (e.g., by adjusting the geometry of the air holes) helps minimize scattering loss and improve the confinement of the guided light.

For the nano-core component, the primary losses are scattering loss ($SL_{S}$) and absorption loss ($AL_{S}$). Rayleigh scattering ($SL_{S}$) is a significant factor due to the large surface area of the solid core, and this can be minimized by improving the surface quality (e.g., reducing roughness and dimensional fluctuations). Material absorption losses $AL_{S}$ can be effectively controlled by selecting higher-purity materials for the nano-core, reducing the losses associated with impurities.

Therefore, the optimal total loss of the NPNANF fiber is achieved by simultaneously optimizing both the hollow-core loss $f\left(r\right)$ and the solid nano-core loss $g\left(r\right)$. The geometric and material properties of both sections must be carefully balanced to minimize the overall loss and maximize transmission efficiency.

To validate the observed asymmetric resonant features, the transmission spectra were fitted with the standard Fano profile:

(12) $$T\left(\omega \right)=T_{0}+\frac{(q+\epsilon {)}^{2}}{1+\epsilon^{2}},\epsilon =\frac{\omega -\omega_{0}}{\frac{\Gamma }{2}}$$

where q is the Fano parameter; $\omega_{0}$ the resonance frequency, and Γ the linewidth. Simulations for nano-core diameters of 0.4 μm and 0.6 μm exhibit clear asymmetric dips, confirming Fano-like interference due to coupling between the localized core mode and anti-resonant cladding resonances.

In our design, the diameter of each air hole is approximately 20% of the total fiber diameter, and the holes are arranged in a densely packed hexagonal lattice to minimize interaction between the light field and tube walls, thereby reducing confinement loss. Additionally, the cladding consists of double annular layers, where the outer air holes are larger than the inner ones. This creates a gradually expanding waveguide profile, enhancing the anti-resonance guiding effect. The detailed parameters, including the diameter of the air hole *d*_1_ ~9.8 μm, inner air hole diameter *d*_2_ ~1.8 μm, the outer diameter of the cladding ring *D* ~44 μm, and the wall thicknesses *t* ~1 μm, are listed in Table 1.

**Figure 5:**
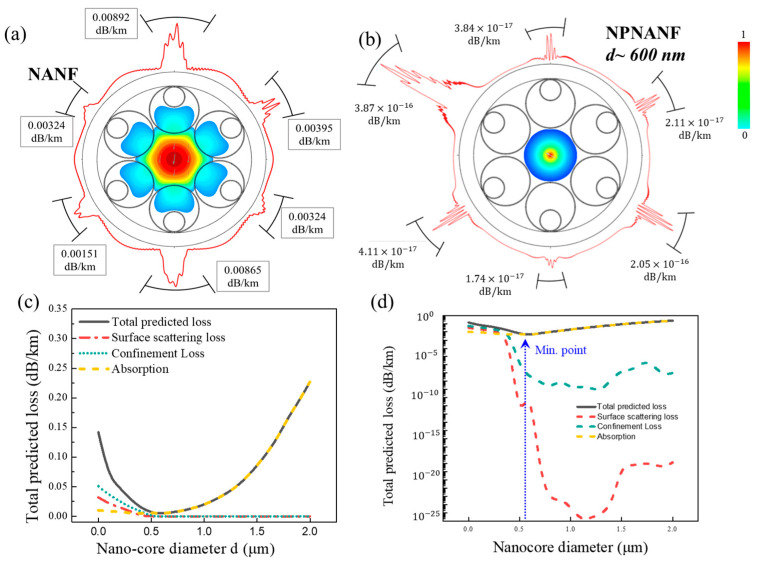
Energy field diagrams showing the scattering loss for (**a**) NANF and (**b**) NPNANF with d ~0.6 μm. Different types of loss change as a function of central nano-core diameter in (**c**) linear scale and (**d**) logarithmic scale for NPNANF.

The Finite Element Analysis (FEA) method was employed to simulate the transmission loss of both NANF and NPNANF across different nano-core diameters. Results are presented in Figure 5.

It is seen from Figure 5a,b that, with the benefit of the solid nano-core in NPNANF, more light field energy is bound in the center of the nano-core when the nano-core diameter is increased. In this way, the total loss of the fiber could be reduced maximally. However, in the design process of NPNANF, the total transmission loss in the solid nano-core and the confinement loss in the air holes of the fibers should be balanced. Since the total loss of NPNANF is more sensitive to the diameter change in the central solid nano-core, the nano-core diameter should be carefully designed and optimized. From Figure 5c,d, it is seen that the diameter of nano-core impacts the total loss in two ways: increasing the diameter of solid nano-core would encounter larger absorption and scattering loss from its large core surface, whereas the confinement loss caused by the air holes is reduced drastically due to the fact that more energy is confined in the center of solid nano-core without relying on anti-resonance effect. From the results presented in Figure 5, it is observed that the optimal nano-core diameter lies around 600 nm, where the minimal total transmission loss is obtained.

For better visualization, Table 3 presents the total loss comparison between the proposed NPNANF and NANF with the same structure.

As shown in Table 3, the proposed NPNANF exhibits several significant advantages. When comparing structures of the same overall size, the insertion of a central solid nano-core into the traditional NANF design can drastically reduce the total transmission loss. This improvement is primarily due to the ability of the high-refractive-index nano-core to confine the optical mode more tightly within the core region, thereby suppressing radiation and leakage losses at the cladding boundaries.

Furthermore, this design enables efficient electromagnetic field confinement within the nano-core. However, if the core diameter is reduced beyond a certain threshold, the coupling between the solid core mode and the anti-resonant hollow-cladding modes becomes more pronounced. In such cases, light propagation occurs not only in the nano-core but also within the surrounding anti-resonant structure, resulting in a hybrid multimodal transmission regime.

This unique propagation behavior further strengthens the anti-resonance effect and helps suppress higher-order modes, thus significantly enhancing the overall transmission efficiency and stability of the fiber.

### 3.3. Loss Calculation and Optimization Results

To provide practical design guidance for the fabrication and performance optimization of the proposed NPNANF structure, a comprehensive parametric study was conducted to investigate the influence of the nano-core diameter on the fiber’s key optical characteristics, including total transmission loss, effective mode area ($A_{eff}$), and modal confinement behavior.

(13) $$A_{eff}=\frac{{\left(\int |E{|}^{2}dS\right)}^{2}}{\int |E{|}^{4}dS}$$

with integration over the entire cross-section, including air regions and cladding. This ensures accurate modeling of the hybrid guidance structure.

**Figure 6:**
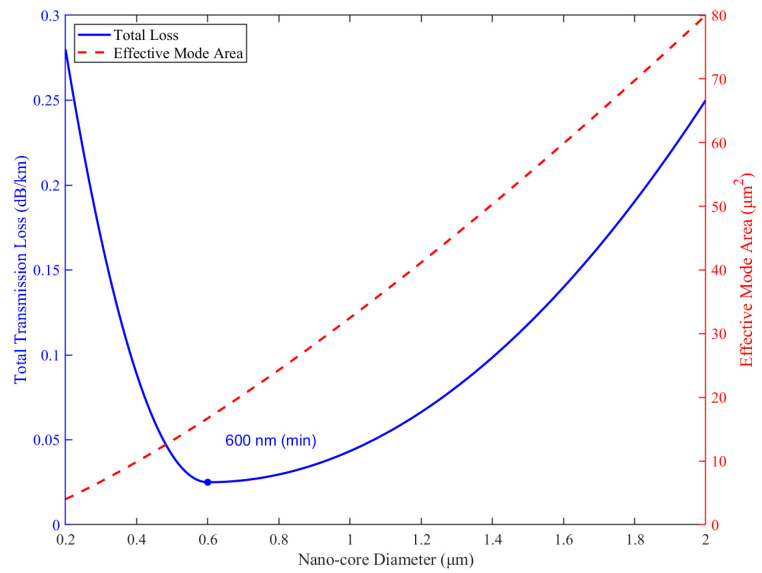
Transmission Loss Trend vs. Core Diameter. Transmission loss (red solid line, left y-axis) and effective mode area (blue dashed line, right y-axis) versus nano-core diameter. An optimal balance between low-loss and strong confinement is achieved at a diameter of ~600 nm.

Figure 6 illustrates the dual dependence of transmission loss and mode area on the nano-core diameter in the range of 0.2–2.0 μm. As shown, the total transmission loss (red solid line, left y-axis) exhibits a characteristic U-shaped trend. When the core diameter is small (<0.4 μm), severe leakage into the cladding and anti-resonant regions results in high confinement loss. As the diameter increases, optical energy becomes more tightly confined within the solid nano-core, significantly reducing leakage. A minimum transmission loss of approximately 0.025 dB/km is observed at a core diameter of 600 nm. Beyond this point, further increasing the core size leads to a gradual increase in loss due to enhanced surface scattering and bending sensitivity associated with larger mode areas.

In parallel, the effective mode area (blue dashed line, right y-axis) increases monotonically with the core diameter. This behavior indicates a more extended modal field as the nano-core expands, which may benefit high-power delivery by mitigating nonlinear effects. However, a larger mode area also reduces modal confinement and may introduce multimode behavior if the core becomes excessively large.

To further quantify the performance trade-offs, Table 4 summarizes the simulated values of total loss, effective refractive index, mode area, and the power confinement ratio across a range of core diameters. The results clearly demonstrate that the proposed NPNANF achieves optimal performance when the nano-core diameter is tuned to approximately 600 nm. At this configuration, the loss is minimized while maintaining a high-power confinement ratio (>92%) and a moderate mode area (22.3 μm^2^), making it highly suitable for both long-haul low-loss transmission and single-mode stability.

To further validate the effectiveness of the proposed NPNANF design, a comparative analysis was conducted between the conventional NANF and the NPNANF with an optimized core diameter of 600 nm. The numerical results are summarized in Table 3, where total transmission loss and effective refractive index at 1550 nm are presented.

As seen in Table 4, the NPNANF exhibits a remarkably low transmission loss of 0.025 dB/km, compared to 12.98 dB/km for the conventional NANF with an identical cladding structure but no central solid core. This represents a more than 500-fold reduction in optical loss. Moreover, the effective refractive index of the NPNANF is slightly higher (1.003 vs. 0.99821), indicating tighter modal confinement within the central solid core.

For conventional NANF without a solid nano-core, the optical field spreads widely across the hollow cladding, resulting in a much larger effective mode area. By contrast, the nano-core of NPNANF tightly confines the optical field, yielding a smaller and more stable mode area.

These results confirm that embedding a high-index nano-core effectively suppresses light leakage, improves confinement, and stabilizes single-mode propagation. The dramatic difference in transmission loss clearly demonstrates the practical advantage of the proposed design for long-haul communication systems and high-power laser delivery, where minimizing optical loss and maintaining mode purity are critical.

Overall, this optimization strategy provides practical guidance for fiber designers seeking to tailor anti-resonant fiber performance to meet the stringent demands of modern photonic systems.

To place our results in context, Table 5 compares the proposed NPNANF with previously reported AR-HCF and NANF designs.

## 4. Conclusions

In this work, we have proposed and numerically validated a novel fiber design: the Nano-Core Plus Node-Free Anti-Resonant Hollow-Core Fiber (NPNANF). By embedding a solid nano-core within a six-tube anti-resonant cladding structure, the proposed fiber achieves an optimal balance between low transmission loss and stable mode confinement. Using finite element analysis (FEA) and beam propagation method (BPM), we systematically investigated the impact of the nano-core diameter on transmission loss, effective refractive index, mode area, and power confinement. The results revealed that a 600 nm core diameter yields a minimum total loss of 0.025 dB/km, representing a >99.8% reduction compared to traditional hollow-core structures with identical cladding geometry. At this optimal point, the effective mode area is also moderate (~22.3 μm^2^), and more than 92% of the optical power is confined within the core. Moreover, the NPNANF structure demonstrates strong resistance to bending loss and a smooth modal profile, making it highly suitable for real-world deployment in scenarios such as long-distance low-loss optical transmission, high-power laser delivery, and nonlinear optical systems. The parametric optimization framework provided in this study offers practical guidance for tailoring anti-resonant fiber performance through precise geometric control of the nano-core.

In conclusion, we have proposed and numerically validated a novel Nano-Core Plus Node-Free Anti-Resonant Hollow-Core Fiber (NPNANF). By embedding a solid nano-core within a six-tube cladding, this design achieves an optimal trade-off between ultra-low transmission loss (0.025 dB/km), strong confinement (>92% power ratio), and bending resistance. These results confirm the suitability of NPNANF for long-distance optical communication, high-power laser delivery, and nonlinear photonics.

Despite these advantages, limitations exist: fabrication of sub-micron nano-core diameters remains challenging, environmental perturbations may influence long-term stability, and experimental validation is required beyond simulations. Addressing these aspects will be the focus of our future work.

## Figures and Tables

**Table 1:** Geometrical structure parameters of NPNANF.

Fiber Type	Diameter of the Nano-Core *d* (nm)	d1/d	d2/d	D/d
NPNANF	600	16.3	3	73.3

**Table 2:** Summary of bending loss performance of HC-based fibers.

Year	Bend Loss	Reference
2018	1 dB/km for R = 10 cm@1512 nm	[11]
2019	15 dB/km for R = 1 cm@1550 nm	[20]
2020	3 dB/km for R = 7 cm@1550 nm	[21]
2022	0.05 dB/km for R = 20 cm@1550 nm	[19]

**Table 3:** Loss comparison between NANF and NPNANF at 1550 nm.

Fiber Type	Diameter of the Nano-Core *d* (nm)	Loss (dB/km)	Effective Refractive Index
NPNANF	600	0.025	1.003
NANF	N/A	12.98	0.99821

**Table 4:** Extended comparison of loss, mode area, and confinement efficiency at 1550 nm.

	Core Diameter (nm)	Loss (dB/km)	Effective Index	Mode Area (μm^2^)	Core Power Ratio (%)
NPNANF	200	1.88	1.0018	72.1	51.2
NPNANF	400	0.24	1.0026	37.5	76.9
NPNANF	600	0.025	1.003	22.3	92.4
NPNANF	1000	0.15	1.0042	11.9	98.1
NANF	—	12.98	0.99821	>100	<5.0

**Table 5:** Comparison of transmission performance between this work and previous studies.

Fiber Type	Wavelength (nm)	Loss (dB/km)	Effective Mode Area (µm^2^)
HC-PBGF	1550	~1.2	>80
Kagome HCF	1550	≥50	~60
NANF	1550	0.22–1.3	50–100
DNANF	1550	0.174	~30
NPNANF (this work)	1550	0.025	22.3

## Data Availability

The data supporting the findings of this study are available from the corresponding authors upon reasonable request.
